# Valve-Sparing Versus Composite Graft Aortic Root Replacement in Acute Type A Aortic Dissection with Standardized Hemiarch Repair: A Propensity Score Matching Analysis

**DOI:** 10.3390/jcdd13030116

**Published:** 2026-03-04

**Authors:** Mohammed Morjan, Tong Li, Luis J. Vallejo Castano, Carlos A. Mestres, Amin Thwairan, Freya S. Jenkins, Hannan Dalyanoglu, Artur Lichtenberg

**Affiliations:** 1 Department of Cardiac Surgery, Medical Faculty, Heinrich Heine University, 40225 Düsseldorf, Germany; mohammed.morjan@med.uni-duesseldorf.de (M.M.); drlitong0601@gmail.com (T.L.); amin.thwairan@med.uni-duesseldorf.de (A.T.); freyasophie.jenkins@usz.ch (F.S.J.); hannan.dalyanoglu@med.uni-duesseldorf.de (H.D.); artur.lichtenberg@med.uni-duesseldorf.de (A.L.); 2 Department of Cardiothoracic Surgery and The Robert WM Frater Cardiovascular Research Centre, University of the Free State, Bloemfontein 9301, South Africa; carlosamestres@gmail.com; 3 Cardiovascular Research Institute Düsseldorf (CARID), Medical Faculty, Heinrich Heine University, 40225 Düsseldorf, Germany

**Keywords:** acute type A aortic dissection, David I procedure, Bentall–De Bono operation

## Abstract

Objectives: The aim of this study was to compare the short- and long-term results of patients who had received aortic valve-sparing reimplantation (David I procedure) vs. aortic root replacement using a composite graft combined with a hemiarch replacement for acute type A aortic dissection (ATAAD) in a propensity score matching analysis. Methods: In this retrospective study we compared the outcomes before and after propensity score matching of patients who underwent emergency surgical repair for ATAAD requiring replacement of the aortic hemiarch with replacement of the aortic root between 2001 and 2023 at our institute. The 154 patients were divided into two groups: the first group consisted of patients undergoing David (n = 59), and the second group of patients undergoing Bentall (n = 95) procedures combined with an aortic hemiarch replacement. To reduce the confounding impact of pre-operative variables in this non-randomized study, 1:1 propensity score matching using the Nearest-Neighbour Matching algorithm was used. Results: Patients in the David plus Hemiarch group were significantly younger (62.16 ± 12.35 vs. 55.55 ± 10.80, *p* = 0.001). After the propensity score matching there were no significant differences between the two groups regarding intra-operative variables and hospital outcomes. In-hospital death was 15% (n = 6) in the David plus Hemiarch group compared to 24% (n = 10) in the Bentall plus Hemiarch group (15% vs. 24%, *p* = 0.40). Operation time was also similar between the two groups, being 402 and 384 min, respectively. Survival analyses also did not show any difference in long-term survival between both groups. Conclusions: When a standardized hemiarch replacement was used, no significant differences in short- and long-term outcomes were observed between a valve-sparing procedure and composite graft replacement in patients undergoing surgical repair for ATAAD. Surgeons should opt for the surgical strategy they are most comfortable with. This study represents one of the few analyses comparing the David and Bentall techniques in ATAAD patients undergoing standardized hemiarch replacement. Despite its retrospective nature, it provides clinically relevant insights for surgical decision-making in emergency settings.

## 1. Introduction

Acute aortic dissection Type A (ATAAD) is a disease associated with a high morbidity and mortality, requiring surgery as the gold standard of therapy. In patients undergoing surgical repair for acute type A aortic dissection, aortic root replacement using a composite graft (Bentall procedure) remains more used than aortic valve-sparing reimplantation (David I procedure). Despite being technically more demanding, younger patients profit more from a valve-sparing procedure than a composite graft technique as it avoids the long-term complications resulting from prosthetic heart valves and oral anticoagulation [1]. In patients with an entry in the inner curvature of the aortic arch or arch branch vessel dissection without cerebral malperfusion syndrome, it is common to perform a hemiarch replacement [2,3]. Recent multicenter registry data and meta-analyses suggest the superiority of the David procedure over the Bentall procedure in terms of long-term survival and freedom from valve-related reintervention in patients with ATAAD involving the aortic root [1,4]. However, these findings often do not account for cases requiring concomitant arch repair, which may influence surgical decision-making and outcomes. The aim of this study was to retrospectively compare the short- and long-term results of patients who received aortic valve-sparing reimplantation vs. aortic root replacement using a composite graft combined with a hemiarch replacement for acute type A aortic dissection using a propensity score matching analysis. While previous studies have compared the David and Bentall procedures in ATAAD, our study is among the first to systematically evaluate these techniques in combination with standardized hemiarch replacement. This approach allows for a more nuanced assessment of outcomes in a real-world emergency surgical context.

## 2. Methods

### 2.1. Study Design

In this retrospective single-centre study, we analyzed all patients who underwent emergency surgery for ATAAD between 2009 and 2023. We further selected patients who received a standardized replacement of the aortic root combined with the aortic hemiarch, and divided them into two groups: patients who received a valve-sparing aortic replacement (Group A), and 95 patients who received an aortic root replacement using a composite graft (Group B). Patients with infective endocarditis, chronic aortic type A dissection or who had received previous cardiac surgery were excluded from this study. The decision to perform a David or Bentall procedure was based on intraoperative assessment of aortic root anatomy, valve morphology, and surgeon experience. David was preferred in younger patients with pliable cusps and isolated aortic regurgitation, whereas Bentall was chosen in cases with calcified valves or mixed lesions, or when valve preservation was not feasible. In the David group, details such as annuloplasty ring sizing, cusp plication, commissural orientation, and annulus/STJ diameters were not consistently documented. In the Bentall group, the type of prosthesis (mechanical vs. biological) and anticoagulation regimen were recorded but not analyzed in detail. Intraoperative and postoperative data from the two groups were compared before and after propensity score matching. To reduce the confounding impact of pre-operative variables in this non-randomized study, 1:1 propensity score matching using the Nearest-Neighbour Matching algorithm was used, leaving 41 patients in each group. Early mortality was defined as all-cause mortality at 30 days, in accordance with the guidelines of The Society of Thoracic Surgeons. Patients undergoing extended arch repair were excluded to ensure a homogeneous study population and to isolate the impact of root replacement technique on outcomes. This design choice enhances internal validity but limits applicability to more complex ATAAD cases.

### 2.2. Ethics

The study was approved by the local ethics committee (Heinrich Heine University Düsseldorf, Protocol #2023-2566, 6 December 2023) and conducted in accordance with the Declaration of Helsinki. Due to the retrospective and anonymized design, patient consent was waived.

### 2.3. Definitions

Acute type A aortic dissection was defined as per the 2024 European Association for Cardio-Thoracic Surgery (EACTS) and Society of Thoracic Surgeons (STS) Guidelines for diagnosing and treating acute and chronic syndromes of the aortic organ [5].

Hemiarch replacement was defined as a resection of the concavity of the aortic arch to the proximal descending thoracic aorta without arch vessel re-implantation, as per the expert consensus document of the European Association for the Cardio-Thoracic surgery (EACTS) and the European Society for Vascular Surgery (ESVS) [6].

Stroke was defined as per Valve Academic Research Consortium (VARC) definitions [7].

### 2.4. Surgical Strategy

With the change in leadership at our institution in 2009, the surgical approach to ATAAD was largely standardized. The right subclavian artery is the preferred site for arterial cannulation, and bilateral antegrade cerebral perfusion under moderate hypothermia (26 °C) is the standard approach for cerebral protection. Intraoperative inspection of the aortic arch to identify potential entry tears is a mandatory step. Hemiarch replacement is performed only in patients without entry tears in the aortic arch or supra-aortic vessels and without aneurysmal changes in the aortic arch. Hemiarch replacement was performed by resecting the entire concavity of the aortic arch just proximal to the origin of the brachiocephalic trunk, extending to the proximal descending thoracic aorta, in a manner similar to the current recommendations from relevant specialty societies [6].

Both the David and Bentall procedures were performed in accordance with the original techniques [8,9]. The decision to proceed with aortic root replacement adhered to the current guideline recommendations throughout the study period. Generally, the indication for aortic root replacement is considered if there is significant valve pathology, if more than one-third of the root circumference is involved in dissection, in the presence of root dilation, or if the coronary arteries are dissected.

### 2.5. Statistics

All statistical analyses were performed using IBM SPSS Statistics Version 26 (IBM Corp., Armonk, NY, USA) and R version 4.3.2 (R Foundation for Statistical Computing, Vienna, Austria). Data were explored for outliers and normality using normal Q-Q plots. Categorical variables are presented as frequencies and percentages. Continuous variables are expressed as mean with standard deviation or median with interquartile range (IQR). To compare data from the David and Bentall combined with hemiarch replacements groups, Pearson’s χ^2^ test (or Fisher’s exact test) was used to compare categorical variables and unpaired Student’s *t*-test (or Mann–Whitney U test) was used to compare continuous variables. To reduce the confounding impact of pre-operative variables in this non-randomized study, 1:1 propensity score matching using the Nearest-Neighbour Matching algorithm was used. The variables used for the matching strategy were age, sex, BSA, emergency OP, DM, CPR, MI, syncope, shock, hypertension and coronary heart disease, as well as a 0.1 Calliper. Variables such as coronary involvement, visceral malperfusion, and the extent of dissection were not consistently documented and were therefore not included in the matching model. Aortic valve pathology, including severity of regurgitation or stenosis, was not included in the propensity score model. Future analyses should incorporate valve lesion severity and perform balance diagnostics such as standardized mean differences and Love plots to ensure comparability. This represents a limitation in baseline comparability. The year of surgery was not included in the matching model. Given the evolution of surgical techniques and perioperative care over the 14-year study period, this may have influenced outcomes. *p*-values < 0.05 were considered statistically significant. Long-term survival was analyzed using Kaplan–Meier curves and Fine–Gray analysis.

## 3. Results

Between 2009 and 2023, 510 patients underwent emergency surgery for ATAAD. Out of these patients, 154 patients received a standardized replacement of the aortic root combined with the aortic hemiarch, forming the study cohort. Fifty-nine patients (38.3%) underwent the David I procedure (Group A), while 95 patients (61.7%) received the classic Bentall–De Bono procedure (Group B).

### 3.1. Patient Characteristics and Preoperative Data

The pre-matching clinical characteristics of the 154 patients receiving a standardized replacement of the aortic root combined with the aortic hemiarch, out of the 510 patients receiving emergency surgery for ATAAD, is presented in Table 1. A total of 59 patients received a David plus Hemiarch procedure, and 95 patients received a Conduit plus Hemiarch procedure. The average age in the David plus Hemiarch group was 55.55 ± 10.80 vs. 62.12 ± 12.35 in the Conduit plus Hemiarch group, and 18.64% of the patients were female in the David plus Hemiarch group compared to 26.32% in the Conduit plus Hemiarch group. A total of 15.79% of the patients who underwent a Conduit plus Hemiarch operation suffered from a syncope preoperatively compared to 0% from the David and Hemiarch group. All the patients in the David plus Hemiarch group suffered from preoperative aortic valve regurgitation, as opposed to 29.47% in the Conduit plus Hemiarch group. Other forms of malperfusion, such as renal, limb, or visceral ischemia, were not systematically recorded in the dataset and could not be included in the analysis. This limits comparability with recent registry data. Emergency surgery was performed on 98.31% of the patients in the David plus Hemiarch group as opposed to 86.32% of the Conduit plus Hemiarch group.

After a 1:1 propensity score matching using the Nearest-Neighbour matching algorithm, there were a total of 41 patients in each group. the results are presented in Table 2. The preoperative laboratory results were also comparable amongst both groups after propensity score matching, and are listed in Table 3.

### 3.2. Perioperative Outcomes

The total length of surgery and the total cardiopulmonary bypass (CPB) time were not significantly longer in group A compared to group B (402.49 ± 104.54 vs. 384.63 ± 121.83, *p* = 0.478) and (289.18 ± 82.13 vs. 267.64 ± 85.16, *p* = 0.340). Aortic cross-clamp time was significantly higher in group A (188.46 ± 52.98 vs. 141.86 ± 56.18, *p* = 0.002). No difference in the duration of Hypothermic Circulatory Arrest (HCA) was observed (29.54 ± 14.67 vs. 31.89 ± 14.23, *p* = 0.544).

Within group B, 36.6% (N = 15) of patients received a mechanical conduit, and 63.4% (N = 26) of patients received a biological conduit. In-hospital mortality was comparable between both groups (14.63% vs. 24.39%, *p* = 0.404). No significant differences in perioperative outcomes were observed between both groups (Table 4).

### 3.3. Follow-Up

**Figure 1:**
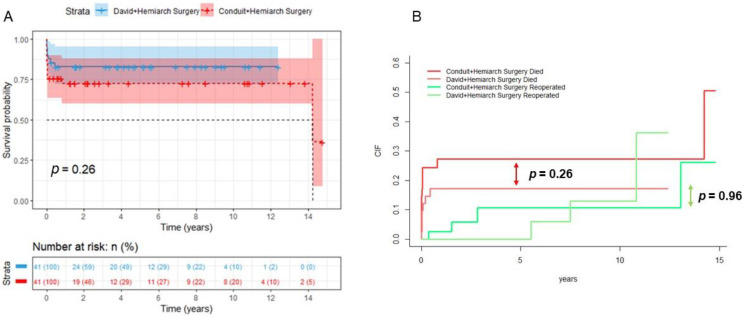
(**A**) Kaplan–Meier survival analysis showing no significant difference in survival between the matched groups at any timepoint (*p* = 0.26). (**B**) Cumulative incidence of reoperation due to valve-related or distal aortic events, analyzed using Fine–Gray competing risk methodology. No significant difference in reoperation rates was observed between the groups (*p* = 0.96).

The maximal follow up of this study was 14 years, with a mean follow-up of 4 ± 4.3 years. Survival analysis showed no significant difference in survival between the David plus Hemiarch group and the Conduit plus Hemiarch group at any timepoint (*p* = 0.26, Figure 1A). Regarding reoperation rates between both groups, the Fine–Gray analysis showed no significant differences (*p* = 0.96, Figure 1B). Echocardiographic follow-up data regarding long-term valve function were not consistently available and therefore were not included in this analysis. This represents a limitation in evaluating the durability of valve-sparing procedures. Long-term bleeding and thromboembolic events were not systematically recorded and are therefore not reported. This limits the evaluation of valve-related complications.

## 4. Discussion

This study employed 1:1 propensity score matching using the Nearest-Neighbour algorithm to compare short- and long-term outcomes between the David and Bentall techniques combined with standardized hemiarch replacement in patients undergoing surgical treatment for acute type A aortic dissection (ATAAD). By focusing on this specific surgical constellation, our analysis contributes meaningfully to the existing literature, offering a differentiated perspective on root and arch management in the emergency setting.

The combination of root replacement with hemiarch repair has not been extensively studied, particularly in the context of ATAAD. Our findings provide practical insights into surgical decision-making and support individualized strategy selection based on anatomical and clinical factors. Notably, the results align with previous studies comparing David and Bentall procedures in settings without arch involvement in both emergent and elective cases [1,10,11].

Recent multicenter registry data and meta-analyses suggest that the David procedure is superior to the Bentall procedure in terms of long-term survival and freedom from valve-related reintervention in patients with ATAAD involving the aortic root. Mosbahi et al. (2019) reported lower in-hospital mortality (2% vs. 8%), higher midterm survival (98.8% vs. 81.3%), and superior freedom from valve-related reintervention (100% vs. 94.6%) in the David group [1]. Similarly, Biancari et al. (2024) analyzed 3735 patients in the ERTAAD registry and found significantly lower 10-year mortality in the David group (30.1% vs. 45.6%; *p* = 0.004) and higher incidence of postoperative dialysis in the Bentall group (17.4% vs. 7.0%; *p* = 0.016) [4]. After propensity score matching, the differences were less pronounced, but the David procedure still showed a trend toward improved long-term outcomes [4].

The Bentall procedure remains widely used due to its technical simplicity and reproducibility. However, the David procedure offers distinct advantages, particularly in younger patients, by preserving native valve function and avoiding lifelong anticoagulation. In our cohort, the David procedure was performed by experienced surgeons in carefully selected patients with pliable valve cusps and favourable root anatomy.

Despite its complexity, the David procedure did not result in longer operative times compared to Bentall (402 vs. 384 min), supporting its feasibility, even in emergency settings, when performed by specialized teams. Nevertheless, the intraoperative decision-making process introduces potential selection bias. Although propensity score matching was applied to mitigate this, residual confounding cannot be fully excluded and should be addressed in future prospective studies.

Pre-matching data (Table 1) reflect a clear selection trend: patients undergoing the David procedure were significantly younger (55.55 ± 10.80 vs. 62.12 ± 12.35 years) and had fewer comorbidities. Variables such as recent myocardial infarction and history of stroke, although not statistically significant, suggest a healthier baseline in the David group. This underscores the importance of incorporating more granular clinical parameters—such as malperfusion syndromes and the extent of dissection—into future matching models.

Perioperative outcomes were comparable between groups. In-hospital mortality was 14.6% in the David group and 24.3% in the Bentall group (*p* = 0.404), aligning with the 16.9% 30-day mortality reported by the GERAADA registry [12]. The higher mortality in the Bentall group may reflect differences in surgeon experience or patient complexity. Notably, postoperative stroke occurred more frequently in the David group (9 vs. 1 patients; *p* = 0.007), a finding that warrants further investigation.

Echocardiographic data—including AR/AS grade, left ventricular dimensions, and postoperative valve function—were not consistently available and could not be analyzed. This represents a major limitation, particularly when evaluating the durability of valve-sparing procedures. A sub-analysis assessing the impact of moderate or severe preoperative aortic regurgitation on outcomes in the David group would be valuable but was not feasible due to incomplete imaging data.

Furthermore, long-term complications such as bleeding, thromboembolic events, and postoperative aortic insufficiency were not systematically recorded. These factors are critical when comparing valve-sparing and valve-replacing strategies and should be addressed in future prospective studies.

The study spans a 14-year period during which surgical techniques, perfusion strategies, and perioperative care have evolved. The year of surgery was not included in the matching model, which may have influenced outcomes. A time-adjusted analysis could help account for these temporal confounders.

Finally, the matched cohort size was relatively small (n = 41 per group), limiting statistical power for subgroup analyses. A post hoc power calculation for key endpoints such as in-hospital mortality, survival, and reoperation should be considered to assess the risk of type II error.

## 5. Limitation

This retrospective single-centre study is subject to inherent limitations, including potential selection bias and limited external validity. The matched sample size was relatively small, reducing statistical power for subgroup analyses. Furthermore, echocardiographic data such as AR/AS grade, left ventricular dimensions, and ejection fraction were not available for all patients and were therefore not analyzed. This limits the assessment of valve-related outcomes. Although follow-up extended up to 14 years, the mean duration was only 4 years, which restricts insights into very-long-term outcomes. Additionally, the exclusion of patients undergoing extended arch repair limits the applicability of the findings to more complex ATAAD cases. Due to the small, matched cohort size, a post hoc power analysis for key endpoints such as in-hospital mortality, survival, and reoperation should be considered to assess the risk of type II error.

## 6. Conclusions

Both the David and Bentall procedures, when combined with standardized hemiarch replacement, demonstrate comparable short- and long-term outcomes in the surgical management of acute type A aortic dissection. While the David procedure offers advantages in selected patients—particularly younger individuals with favourable valve anatomy—its application remains limited by its technical complexity and the need for surgical expertise. Our findings support the feasibility of valve-sparing surgery in emergency settings and highlight the importance of individualized surgical decision-making. Given the limitations in echocardiographic follow-up, long-term valve function, and complication data, further prospective studies are warranted to refine patient selection and assess durability. Surgeons should continue to base their approach on anatomical suitability, clinical urgency, and institutional experience.

## Figures and Tables

**Table 1:** Pre-matching baseline.

Variables	David + Hemiarch (n = 59)	Bentall + Hemiarch (n = 95)	*p* Values
Age (years)	55.55 ± 10.80	62.16 ± 12.35	0.001
Sex (female, %)	11 (18.64)	25 (26.32)	0.330
BMI (kg/m^2^)	27.68 (21.26, 44.75)	27.34 (18.31–37.86)	0.383
BSA (m^2^)	2.15 ± 0.23	2.02 ± 0.22	0.001
NYHA classification			0.375
I	56	89	
II	1	1	
II–III	0	2	
III	0	2	
III–IV	1	1	
IV	1	0	
CCS classification			0.442
0	42	72	
1	2	0	
2	1	3	
3	7	10	
4	7	10	
MI in last 3 months (n, %)	2 (3.39)	8 (8.42)	0.319
Preoperative troponin (mmol/L)	21 (2.7929)	25 (0.627)	0.826
Stroke in history (n, %)	6 (10.17)	14 (14.74)	0.469
Preoperative stroke (n, %)	0	15 (15.79)	0.001
CPR (n, %)	4 (6.78)	11 (11.58)	0.410
Preoperative aortic valve regurgitation (n, %)	59 (100)	28 (29.47)	<0.001
Preoperative aortic valve stenosis (n, %)	0	9 (9.47)	0.013
COPD (n, %)	5 (8.47)	7 (7.37)	1.000
Diabetes (n, %)	6 (10.17)	5 (5.26)	0.336
Hypertension (n, %)	28 (47.46)	65 (68.42)	0.011
Smoking history (n, %)	18 (30.51)	23 (24.21)	0.454

BMI: Body Mass Index; BSA: Body Surface Area; CCS: Chronic Coronary Syndromes; MI: Myocardial infarction; CPR: Cardiopulmonary Resuscitation; COPD: Chronic Obstructive Pulmonary Disease.

**Table 2:** Post-matching baseline.

Variables	David + Hemiarch (n = 41)	Bentall + Hemiarch (n = 41)	*p* Values
Age (years)	56.70 ± 10.81	56.82 ± 11.87	0.960
Sex (female, %)	9 (21.95)	7 (17.07)	0.781
BMI (kg/m^2^)	28.93 ± 5.69	28.71 ± 4.11	0.841
BSA (m^2^)	2.15 ± 0.23	2.10 ± 0.19	0.362
NYHA classification			0.399
I	38	40	
II	1	0	
II–III	0	1	
III	0	0	
III–IV	1	0	
IV	1	0	
CCS classification			0.422
0	28	33	
1	2	0	
2	1	2	
3	4	3	
4	6	3	
MI in last 3 months (n, %)	2 (4.88)	1 (2.44)	1.000
Coronary Heart Disease (n, %)	11 (26.83)	9 (21.95)	0.798
Stroke in history (n, %)	5 (12.20)	5 (12.20)	1.000
preoperative stroke (n, %)	0	0	/
CPR (n, %)	3 (7.32)	3 (7.32)	1.000
Preoperative aortic valve regurgitation (n, %)	41 (100)	12 (29.29)	<0.001
Preoperative aortic valve stenosis (n, %)	0	5 (12.20)	0.055
COPD (n, %)	2 (4.88)	2 (4.88)	1.000
Diabetes (n, %)	5 (12.20)	4 (9.76)	1.000
Hypertension (n, %)	23 (56.10)	22 (53.66)	1.000
Smoking history (n, %)	10 (24.39)	14 (34.15)	0.467

BMI: Body Mass Index; BSA: Body Surface Area; CCS: Chronic Coronary Syndromes; MI: Myocardial infarction; CPR: Cardiopulmonary Resuscitation; COPD: Chronic Obstructive Pulmonary Disease.

**Table 3:** Preoperative laboratory parameters.

Variables	David + Hemiarch (n = 41)	Bentall + Hemiarch (n = 41)	*p* Values
Troponin	25 (2,7929)	26 (0,627)	0.824
Bilirubin	0.64 (0.17,1.90)	0.7 (0.22,1.91)	0.867
Creatine (mg/dL)	1.1 (0.69,2.1)	1.0 (0.5,1.9)	0.051
INR	1.1 (0.9,3.2)	1.1 (0.9,2.3)	0.577
Hb	13.6 (5.2,16.2)	13.2 (6.7,15.7)	0.492
HK	40 (17,47)	40 (21,49)	0.491
Na	139 (130,146)	140 (130,146)	0.709

INR: International Normalized Ratio; Hb: Hemoglobin; HK: Hematocrit; Na: Sodium.

**Table 4:** Intraoperative and perioperative outcomes.

Variables	David + Hemiarch (n = 41)	Bentall + Hemiarch (n = 41)	*p* Values
Operation time (min)	402.49 ± 104.54	384.63 ± 121.83	0.478
CPB time (min)	289.18 ± 82.13	267.64 ± 85.16	0.340
Aortic cross-clamp time (min)	188.46 ± 52.98	141.86 ± 56.18	0.002
HCA time (min)	29.54 ± 14.67	31.89 ± 14.23	0.544
Combined CABG	3	2	1.000
Intraoperative death (n, %)	1	1	1.000
In-hospital death	6	10	0.404
CPR	3	4	1.000
Intraoperative LCOS	0	2	0.494
Postoperative LCOS	2	5	0.432
ECMO support	2	2	1.000
Major bleeding	7	5	0.756
Re-thoracotomy	9	9	1.000
Delir	10	11	1.000
Infections	5	5	1.000
Respiratory failure	10	11	1.000
Gastro-intestinal complication	5	3	0.712
Renal failure	10	12	0.804
Dialysis/CVVHD	9	8	1.000
Stroke	10	4	0.078

CPB: Cardiopulmonary bypass, HCA: Hypothermic Circulatory Arrest, CABG: coronary artery bypass grafting, LCOS: Low Cardiac Output Syndrome, ECMO: Extracorporeal Membrane Oxygenation.

## Data Availability

The data underlying this study were obtained retrospectively from clinical records and contain sensitive personal health information. Due to privacy regulations and ethical considerations, the raw data cannot be made publicly available. Researchers with a legitimate interest may request access to anonymized or aggregated data, subject to approval by the responsible ethics committee and in compliance with applicable data protection laws.
